# Sequential Cisplatin Therapy and Vaccination with HPV16 E6E7L2 Fusion Protein in Saponin Adjuvant GPI-0100 for the Treatment of a Model HPV16+ Cancer

**DOI:** 10.1371/journal.pone.0116389

**Published:** 2015-01-05

**Authors:** Shiwen Peng, Joshua W. Wang, Balasubramanyam Karanam, Chenguang Wang, Warner K. Huh, Ronald D. Alvarez, Sara I. Pai, Chien-fu Hung, T. -C. Wu, Richard B. S. Roden

**Affiliations:** 1 Department of Pathology, The Johns Hopkins University, Baltimore, Maryland, United States of America; 2 Department of Biostatistics, The Johns Hopkins University, Baltimore, Maryland, United States of America; 3 Department of Oncology, The Johns Hopkins University, Baltimore, Maryland, United States of America; 4 Department of Gynecology and Obstetrics, The Johns Hopkins University, Baltimore, Maryland, United States of America; 5 Division of Gynecologic Oncology, University of Alabama at Birmingham, Birmingham, Alabama, United States of America; 6 Department of Biology and Center for Cancer Research, Carver Research Foundation, Tuskegee University, Tuskegee, Alabama, United States of America; 7 Department of Surgery, Massachusetts General Hospital, Boston, Massachusetts, United States of America; Mie University Graduate School of Medicine, Japan

## Abstract

Clinical studies suggest that responses to HPV16 E6E7L2 fusion protein (TA-CIN) vaccination alone are modest, and GPI-0100 is a well-tolerated, potent adjuvant. Here we sought to optimize both the immunogenicity of TA-CIN via formulation with GPI-0100 and treatment of HPV16+ cancer by vaccination after cisplatin chemotherapy. HPV16 neutralizing serum antibody titers, CD4+ T cell proliferative and E6/E7-specific CD8+ T cell responses were significantly enhanced when mice were vaccinated subcutaneously (s.c.) or intramuscularly (i.m.) with TA-CIN formulated with GPI-0100. Vaccination was tested for therapy of mice bearing syngeneic HPV16 E6/E7+ tumors (TC-1) either in the lung or subcutaneously. Mice treated with TA-CIN/GPI-0100 vaccination exhibited robust E7-specific CD8+ T cell responses, which were associated with reduced tumor burden in the lung, whereas mice receiving either component alone were similar to controls. Since vaccination alone was not sufficient for cure, mice bearing s.c. TC-1 tumor were first treated with two doses of cisplatin and then vaccinated. Vaccination with TA-CIN/GPI-0100 i.m. substantially retarded tumor growth and extended survival after cisplatin therapy. Injection of TA-CIN alone, but not GPI-0100, into the tumor (i.t.) was similarly efficacious after cisplatin therapy, but the mice eventually succumbed. However, tumor regression and extended remission was observed in 80% of the mice treated with cisplatin and then intra-tumoral TA-CIN/GPI-0100 vaccination. These mice also exhibited robust E7-specific CD8+ T cell and HPV16 neutralizing antibody responses. Thus formulation of TA-CIN with GPI-0100 and intra-tumoral delivery after cisplatin treatment elicits potent therapeutic responses in a murine model of HPV16+ cancer.

## Introduction

‘High risk’ human papillomaviruses (hrHPV) cause 5.2% of all cancers worldwide [1]. While persistent hrHPV infection is a necessary cause of cancer, the great majority of infections are spontaneously cleared by the host immunity. Secondary prevention via cytologic and HPV screening and intervention programs have reduced the burden of cervical cancer by an estimated 80% in developed countries and now two preventive HPV vaccines target the two most prevalent of the 14 hrHPV types, HPV16 and HPV18. HPV16 is the genotype present in 50–60% of cervical cancer, in 87% of HPV+ oropharyngeal carcinomas [2], in 55% and 76% of HPV+ invasive vaginal and vulva carcinomas [3], and in 73% of anal cancer [4]. The substantial efficacy and safety of licensed HPV vaccines for the prevention of new HPV16 and HPV18 infections is well documented [5]. However, the protection afforded by these commercially available vaccines is generally type restricted [6], and vaccination rates unfortunately remain low in developing countries. Importantly, these vaccines lack therapeutic activity for those patients with persistent HPV infection and established HPV associated cervical dysplasia [7], Therapeutic HPV vaccination has the potential to augment the efficacy of conventional non-specific, surgical and ablative therapies of high grade neoplasia, or even chemoradiation therapy of invasive HPV+ cancers. Despite the use of cisplatin and/or radiation therapy [8], the five-year survival of advanced cervical cancer patients remains <30%. Thus, targeted treatment strategies, such as therapeutic HPV vaccination, are needed to improve outcomes in patients with advanced cervical cancer [9].

The candidate therapeutic HPV vaccine TA-CIN is a recombinant protein comprising a fusion of HPV16 oncoproteins E6, E7 and the minor capsid protein L2 that is purified from *E. coli* [10]. E6 and E7 viral oncoproteins are promising targets for immunotherapy, being expressed in all infected cells, required for the viability of HPV+ cancer cells and absent from normal host cells. The minor capsid protein L2 is not detectably expressed in HPV+ cancer cells, but it is a potential therapeutic antigen for precursor lesions [11], [12], [13]. Furthermore L2 contains conserved neutralizing epitopes and has potential as a broadly prophylactic HPV antigen [14]. Three monthly immunizations of healthy volunteers with TA-CIN without adjuvant, elicited an L2-specific neutralizing serum antibodies and T cell proliferative responses in a dose dependent manner [15]. E6 and E7-specific CD8 T cell immunity was detected by IFNγ ELISPOT in 8 of 11 evaluable subjects at the highest dose, although E6 was the dominant antigen. However, the overall response was modest, suggesting the need for an adjuvant [16], [17] or heterologous boosting with a recombinant viral vector [18], [19], [20].

Protein-based vaccination without an adjuvant typically induces weak immune responses. GPI-0100 is a potent adjuvant derived by modifying selected natural saponins [21] to eliminate relatively toxic and unstable acyl moieties, and introducing a lipophilic moiety [22], [23]. Doses of 100 µg to 5000 µg of GPI-0100 have been tested with several antigens in early phase clinical trials and were well tolerated [21], [24]. Vaccination of macaques with TA-CIN in combination with the adjuvant GPI-0100 (TA-CIN/GPI-0100) was also well tolerated and elicited robust neutralizing antibody titers against HPV16, with lesser responses against other HPV types tested, and T cell responses to HPV16 E6 and E7 [16]. Formulation of TA-CIN with GPI-0100 profoundly enhanced the neutralizing antibody response and protected mice from experimental skin challenge with HPV16 pseudovirions that deliver a luciferase reporter. Inclusion of GPI-0100 also enhanced the E7-specific CD8 T cell response to TA-CIN vaccination and protected mice from challenge with the TC-1 cell line, an HPV16 E6/E7+ murine tumor model [16]. However, its therapeutic activity against established tumor was not tested.

Recent studies suggest that local immunization is important to elicit a cellular immune response that homes back to the relevant site and that chemotherapy can enhance the immune response in part by shrinking disease burden, releasing tumor antigens and transiently modifying the immune microenvironment within the tumor [25], [26], [27], [28], [29], [30], [31]. Treatment with cisplatin and/or low-dose radiation can enhance the potency of therapeutic HPV vaccines in mouse models [32], [33], [34]. Concurrent treatment with cisplatin and intratumoral injection (i.t.) of E7 peptide in C57BL/6 mice bearing E6/E7-expressing TC-1 tumors generated significantly more E7-specific IFN-γ-secreting CD8^+^ T cells and could cure many mice, whereas those treated with either therapy alone did not generate similar tumor growth control. Importantly, subcutaneous administration of E7 peptide controlled tumor growth less effectively than intratumoral administration, indicating that delivery of antigen into the tumor microenvironment enhances priming of a tumor antigen-specific CD8+ T cell immune response following chemotherapy [34]. Cisplatin treatment also transiently induced a significant accumulation of dendritic cells (DCs) in the tumor microenvironment. Furthermore, intratumoral injection of antigenic peptide led to the uptake of the E7 peptide by the CD11c+ DCs and migration of the E7 peptide-loaded DCs to the draining lymph nodes [34]. The E7 peptide-loaded DCs in the draining lymph node could activate E7-specific CD8+ T cells. Importantly, this marked enhancement of E7-specific CD8+ T cells in circulation and antitumor effects was also observed when TC-1 tumor-bearing mice were treated with cisplatin and intratumoral TA-CIN vaccination [35].

Our prior studies in mice and macaques support the safety and potency of GPI-0100 as an adjuvant for TA-CIN, but did not examine its impact upon therapeutic activity [16]. Here we compared vaccination alone and cisplatin followed by intratumoral or intramuscular vaccination TA-CIN formulated with GPI-0100 adjuvant for the treatment of established TC-1 tumors.

## Materials and Methods

### Ethics Statement

The human-derived cell line 293TT is used in this study. This cell line has been previously described [37], [38]. Animal studies were carried out in accordance with the recommendations in the Guide for the Care and Use of Laboratory Animals of the National Institutes of Health and with the prior approval of the Animal Care and Use Committee of Johns Hopkins University (MO14M43). Mice were monitored at least three times per week, and humane endpoints were used in survival studies; mice were euthanized upon excess tumor burden (≥2 cm diameter or ulceration), or first evidence of distress such as loss of weight (≥10%), lethargy, poor coat quality, sensitivity to light, nesting or scratches. Mice were anesthetized with isoflurane inhalation, euthanized by carbon dioxide asphyxiation and death ensured by cervical dislocation.

### Mice

5∼8 weeks old female C57BL/6 or BALB/c mice were purchased from the National Cancer Institute (Frederick, MD) and housed for at least one week prior to study. All mice were maintained at Johns Hopkins University School of Medicine (Baltimore, MD) cancer center animal facility under specific-pathogen free conditions. The mice were maintained 5 per microisolator cage with sterile food and bedding, changed weekly, and randomized by cage.

### Peptides, antibodies and reagents

The H-2K^b^-restricted HPV16 E6aa50-57 peptide, YDFAFRDL, and H-2D^b^-restricted HPV16 E7aa49-57 peptide, RAHYNIVTF were synthesized by Macromolecular Resources (Denver, CO) at a purity of ≥80%. FITC or PE-conjugated anti-mouse CD4 (clone RM4-5) and CD8a (clone 53.6.7), and FITC-conjugated anti-mouse IFN-γ (clone XMG1.2) antibodies were purchased from BD Pharmingen (San Diego, CA). PE-conjugated, HPV16 E7aa49-57 peptide loaded H2-D^b^ tetramers were obtained from the National Institute of Allergy and Infectious Diseases Tetramer Facility. Medroxyprogesterone acetate was purchased from Greenstone LLC (Peapack, NJ), and 4% Nonoxynol-9 (N-9) was purchased from Revive personal products company (Madison, NJ). Cisplatin, Tween-40 and mannitol were purchased from Sigma (St. Louis, MO).

### Cells

HPV-16 E6 and E7-expressing TC-1 cells were generated as previously described [36]. CT26 colon carcinoma cells were obtained from the American Type Culture Collection (Manassas, VA). The cells were maintained in RPMI medium supplemented with 2 mM glutamine, 1 mM sodium pyruvate, 100IU/mL penicillin, 100 µg/mL streptomycin and 10% fetal bovine serum (FBS). 293TT cells were cultured in DMEM medium containing 2 mM glutamine, 1 mM sodium pyruvate, 100IU/mL penicillin, 100 µg/mL streptomycin and 10% FBS [37].

### Vaccine and formulation

TA-CIN has been described previously and was kindly provided by Cancer Research Technology, UK [15]. GPI-0100 was generously provided by Hawaii Biotech Inc. (Aiea, HI). For vaccination studies, TA-CIN (31.25 µg/mL) was formulated with GPI-0100 (250 µg/mL), alone or with either Tween-40 (4 mg/mL), or mannitol (50.7 mg/mL) in 0.025 M sodium phosphate buffer (pH 7.2). To prepare vaccine for intra-tumoral injection, TA-CIN (312.5 µg/mL) was formulated with GPI-0100 (2500 µg/mL) along with mannitol (50.7 mg/mL) in 0.025 M sodium phosphate buffer (pH 7.2). The formulated vaccine was incubated overnight on a gentle shaker at 4°C prior to use. The frozen formulation of TA-CIN/GPI-0100 included mannitol, and after overnight incubation, it was divided into aliquots and frozen using dry ice/isopropanol. The aliquots were then stored at −80°C until use.

### Production of HPV16 and HPV58 pseudovirus and neutralization assay

Pseudoviruses (PsV) were produced by co-transfection of 293TT cells [37], [38] with plasmids encoding HPV L1 and L2 and reporter plasmid expressing either secreted alkaline phosphatase (SEAP) or firefly luciferase [39], [40]. SEAP-based pseudovirus neutralization assays were performed as described previously [38]. Briefly, HPV16 SEAP PsVs were incubated with titrated mouse sera (starting dilution 1∶50) or 4 µL of positive control monoclonal antibody RG-1 (1.1 mg/mL) for 2 hours at 37°C before addition to 293TT cells. The cells were then incubated for 72 hours, and the supernatants were collected. SEAP activity was measured by via the colorimetric assay method. Serum neutralization titers were defined as the highest dilution that resulted in at least 50% reduction of SEAP activity compared to virus only infection controls.

### Intracellular cytokine staining and flow cytometry analysis

To detect HPV16 E6 or E7-specific CD8^+^ T cell responses by IFN-γ intracellular staining, splenocytes were stimulated with either HPV16 E6aa50-57 or E7aa49-57 peptide (1 µg/mL) at the presence of GolgiPlug (BD Pharmingen, San Diego, CA) at 37°C overnight. To detect TA-CIN-specific CD4^+^ and CD8^+^ T cell responses, splenocytes were stimulated with 10 µg/mL of TA-CIN protein for 24 hours at 37°C. GolgiPlug was then added to the cells and further incubated overnight at 37°C. When CT26 cells transfected with HPV16 L2 were used, CT26 cells were first transfected with plasmid encoding codon-optimized HPV16 L2 [41] using Lipofectamine 2000 (Invitrogen, Carlsbad, CA). Splenocytes were then co-cultured with HPV16 L2 transfected CT26 cells with the E∶T ratio of 10∶1 at the presence of GolgiPlug at 37°C overnight. The stimulated splenocytes were then washed once with PBS containing 0.5% BSA and stained with either PE-conjugated anti-mouse CD4 or CD8 antibody. Cells were permeabilized and fixed with Cytofix/Cytoperm kit according to the manufacturer's instruction (BD Pharmingen, San Diego, CA). Intracellular IFN-γ was stained with FITC-conjugated rat antimouse IFN-γ. Flow cytometry analysis was performed using FACSCalibur flow cytometer with CellQuest software (BD biosciences, Mountain View, CA).

### Tetramer staining

For tetramer staining, PBMCs from the mice were stained with purified anti-mouse CD16/32 (Fc block, BD Pharmingen, San Diego, CA) first, and then stained with anti-mouse CD8-FITC, PE-conjugated, HPV16 E7aa49-57 peptide, RAHYNIVTF loaded H2-D^b^ tetramer at 4°C. After wash, the cells were stained with 7-AAD before flow cytometry analysis to exclude dead cells. The cells were acquired with FACSCalibur flow cytometer and analyzed with CellQuest software.

### Passive transfer of mouse or macacque sera and vaginal HPV PsV challenge

For passive transfer studies, groups of female BALB/c mice (n = 5) were injected with 3 mg of medroxyprogesterone subcutaneously four days prior to vaginal PsV challenge. One day before vaginal challenge, 90 µL of sera from each control or TA-CIN vaccinated female BALB/c mice were pooled in their respective groups with an additional 150 µL of PBS (total volume 600 µL). As a positive control, the mouse monoclonal antibody RG-1 was utilized in three separate doses- High (50 µg/mouse), Medium (25 µg/mouse) and Low (12.5 µg/mouse). Subsequently, 100 µL of the respective pooled sera or RG-1 doses was then injected i.p. into each mouse of the respective group. At 24 hours post passive transfer, vaginal challenge with HPV PsV was performed as described previously [42] using ∼10^8^ viral genomic equivalent (VGE) units/mouse. Three days after HPV PsV challenge, the expression of luciferase in the vaginal vault was acquired with a Xenogen IVIS 100 (Caliper Life Science, Hopkinton MA) imager and analyzed with Living Image 2.5 software. The same steps were performed for passive transfer studies utilizing sera from 3 macaques (I.D 746, 811 or 831) either pre- or post- third vaccination with TA-CIN+GPI-0100 generated in a prior study [16]. 100 µL of pre-immune sera (dilution = 1∶200, based on mouse blood volume estimate of 2 mL) or vaccinated sera was titrated with a dilution range of 1∶200-1∶2000 was passively transferred i.p. into groups of naïve mice (n = 5). The following day, the mice were challenged intravaginally with HPV58 PsV and imaged 72 hours thereafter to assess infectivity.

### Treatment studies of hematogeneosuly spread TC-1 tumor cells

C57BL/6 mice were injected with 5×10^4^ of TC-1 cells intravenously. To test the antitumor effect induced by vaccination, 3 days later the mice were vaccinated three times with the indicated regimen. When the untreated mice showed signs of sickness (see ethics statement), all mice were sacrificed, and splenocytes were prepared to detect HPV16 E6 or E7 or TA-CIN-specific CD4^+^ and CD8^+^ T cell responses. Lungs were harvested to check the tumor growth. Alternatively, PBMCs and sera were collected from the mice for the analysis of HPV16 E7-specific CD8^+^ T cell responses with tetramer staining or HPV16-specific neutralizing antibody titer with HPV16-SEAP PsV respectively.

### Treatment studies of subcutaneous TC-1 tumor

1×10^5^ of TC-1 tumor cells were injected subcutaneously into C57BL/6 mice. On day 5 and 12 after tumor cell challenge, mice were injected with 5 mg/kg of cisplatin or saline intraperitoneally. One day after cisplatin or saline injection, mice were left either untreated, or vaccinated with GPI-0100 only, or TA-CIN only, or TA-CIN plus GPI-0100 intratumorally. One group of cisplatin treated mice was vaccinated with TA-CIN plus GPI-0100 intramuscularly. A volume of 200 µL was used for all vaccination studies except 20 µL for intratumor vaccination. The mice were boosted twice with the same regimen. Tumor growth was monitored by visual inspection and measurement of tumor diameter with calipers twice each week. Tumor volume was calculated using the formula [largest diameter × (perpendicular diameter)^2^] ×π/6. Tumor survival was recorded as either natural death or a tumor diameter greater than 2 cm.

### Statistical analysis

Cell mediated immunity data were expressed as mean ± standard deviation (SD). Comparisons between groups utilized Student's *t* test. Survival distributions for mice in different groups were estimated using the Kaplan-Meier method and compared with the log-rank test. For passive transfer experiments, the data was expressed in terms of mean percentage infection ± standard error (SE). A p-value <0.05 was considered statistically significant. Multiplicity adjustment was not considered because of the exploratory nature of the data analysis.

## Results

### GPI-0100 significantly enhances HPV16 E7-specific CD8+ T cell responses and tumor therapy induced by TA-CIN

We have previously demonstrated that formulation of TA-CIN with GPI-0100 greatly enhances both HPV16-specific neutralizing serum antibody titers and E7-specific CD8+ T cell responses to subcutaneous vaccination of naïve mice [16]. To test whether different batches of GPI-0100 and TA-CIN can generate similar data, we vaccinated naïve C57BL/6 mice with two different cGMP batches of TA-CIN (0847FP and 0861FP) formulated with three different cGMP batches of GPI-0100 (0400806, 0400306R and 0400106R) subcutaneously. No significant difference was observed for both HPV16-specific neutralizing antibody titer and E6/E7-specific CD8+ T cell responses (data not shown). Since vaccination route may potentially impact the immune response, we also compared the immunogenicity of TA-CIN formulated with GPI-0100 that was given by either subcutaneous (s.c.) or intramuscular injection (i.m.). No significant difference was observed for either HPV16-specific neutralizing serum antibody titer or E6/E7-specific CD8+ T cell responses (S1 Fig.).

**Figure 1:**
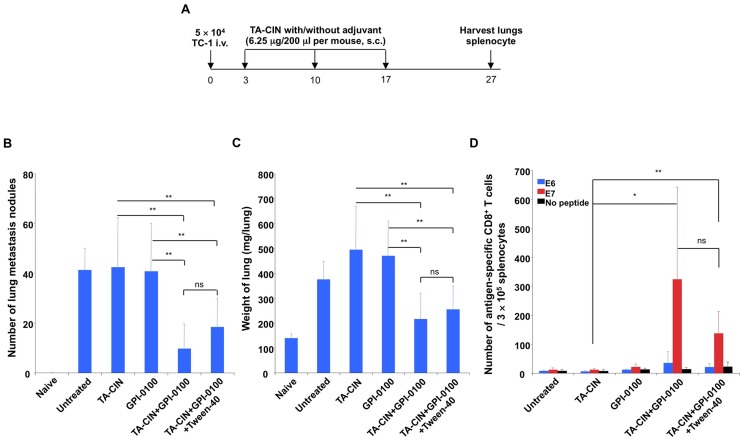
GPI-0100 significantly enhances HPV16 E7-specific CD8+ T cell responses and antitumor effect induced by TA-CIN in the TC-1 lung metastasis model. **A.** Schematic illustration of the experiment design. Briefly, 5∼8 week old female C57BL/6 mice (10 mice/group) were injected with 5×10^4^ TC-1 cells intravenously on day 0. On day 3, the mice were vaccinated with either 6.25 µg/mouse of TA-CIN, or formulated with 50 µg of GPI-0100, or formulated with 50 µg of GPI-0100 in 800 µg of Tween-40 via subcutaneous injection. The mice were boosted twice with the same regimen. On day 27, the mice were sacrificed to harvest lungs and spleens. **B.** Summary of the number of TC-1 metastasis nodules in the lungs of each mouse (and photographs of the lungs are presented in S2A Fig.). **C.** Summary of the weight of the lungs. **D.** Summary of flow cytometry analysis of HPV16 E6 and E7-specific CD8^+^ T cell responses analyzed by IFN-γ intracellular staining. The data were acquired with FACSCalibur, analyzed with CellQuest and representative data is shown in S2B Fig.

In our prior study, vaccination with TA-CIN formulated with GPI-0100 completely protected mice from a subsequent subcutaneous challenge with the HPV16 E6/E7-expressing TC-1 tumor model [16], but treatment of pre-existing and metastatic TC-1 tumor was not tested. Injection of TC-1 cells in the tail vein (i.v.) leads to their establishment in the lungs, providing a model of pulmonary metastatic HPV16+ cancer [43]. Therefore we tested whether formulation of TA-CIN with GPI-0100 impacts the therapeutic effect against TC-1 tumor implants in the lungs. The TC-1 tumor cells were injected in the tail vein of C57BL6 mice and 3 days later, the tumor-bearing mice were vaccinated subcutaneously with either a low dose (6.25 µg/mouse) of TA-CIN alone or formulated with 50 µg GPI-0100, followed by two boosters at one week intervals. Lungs were harvested ten days later to evaluate tumor growth (Fig. 1A). Neither vaccination with TA-CIN alone, nor GPI-0100 alone inhibited TC-1 tumor growth in the lungs. In the contrast, mice vaccinated with TA-CIN formulated with GPI-0100 had significantly fewer pulmonary tumor nodules and therefore a lower lung weight compared to unvaccinated mice (Fig. 1B and C and S2A Fig.). However, the tumor burden was only reduced, not completely eliminated. When HPV16 E6/E7-specific CD8+ T cell responses were analyzed, we found that TC-1 tumor-bearing mice vaccinated with TA-CIN formulated with GPI-0100 induced dramatically higher E7-specific CD8+ T cell responses than either TA-CIN or GPI-0100 only (Fig. 1D and S2B Fig.). Although TA-CIN also contains an E6 CD8+ T-cell epitope, only weak E6-specific CD8+ T cell responses were observed as E7 is the dominant antigen in C57Bl/6 mice (Fig. 1D and S2B Fig.). Formulation with Tween-40 has been proposed to boost the adjuvant effect of GPI-0100 [44]. However, consistent with our previous findings in naïve mice [16], inclusion of Tween-40 in TA-CIN/GPI-0100 formulation did not significantly impact the anti-tumor effect or E6/E7-specific CD8+ T cell responses (Fig. 1D and S2B Fig.). Taken together, these data extend our previous finding that GPI-0100 is able to significantly enhance the immunogenicity of TA-CIN including an E7-specific CD8+ T cell response and here show its potential for therapeutic activity against HPV16-transformed tumor in C57BL6 mice.

### Immunogenicity of TA-CIN formulated with GPI-0100 in mannitol and frozen

**Figure 2:**
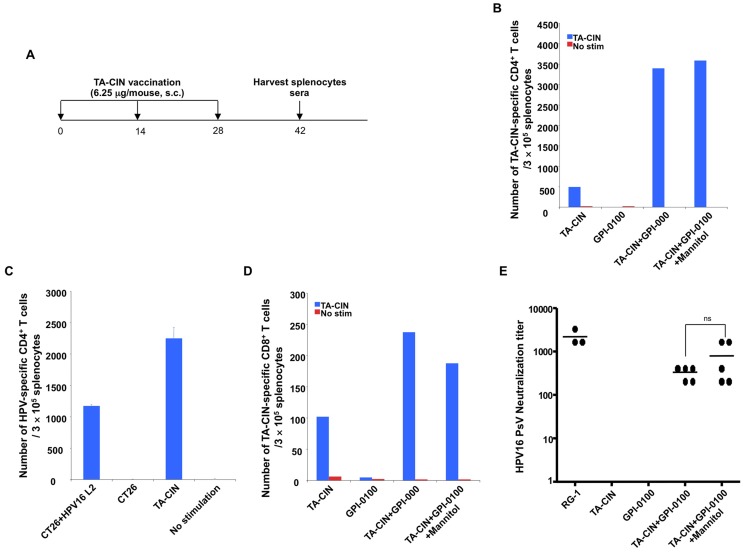
Effect upon HPV16-specific T cell and neutralizing antibody responses to vaccination with TA-CIN/GPI-0100 after a freeze/thaw cycle. **A.** Schematic illustration of the experimental protocol. Briefly, 5∼8 weeks old female BALB/c mice (5 mice/group) vaccinated subcutaneously with either 6.25 µg/mouse of TA-CIN, or formulated with 50 µg of GPI-0100, or formulated with 50 µg of GPI-0100 in 50.7 mg/mL of mannitol and subjected to a single freeze/thaw cycle. The mice were boosted twice with the same regimen with 2-week interval. Two weeks after the last vaccination, sera and splenocytes were harvested. **B.** Summary of the flow cytometric analysis of TA-CIN-specific CD4^+^ T cell responses analyzed by IFN-γ intracellular staining (and representative data is shown in S3A Fig.). **C.** Summary of the flow cytometry analysis examining HPV16-specific CD4^+^ T cell responses induced by TA-CIN formulated with GPI-0100 in mannitol and frozen/thawed once. Splenocytes were harvested after vaccination and stimulated with either TA-CIN or HPV16 L2 transfected CT26 cells (and representative data is shown in S3B Fig.). **D.** Summary of the flow cytometry analysis of TA-CIN-specific CD8^+^ T cell responses analyzed by IFN-γ intracellular staining. The data were acquired with FACSCalibur and analyzed with CellQuest (and representative data is shown in S3C Fig.). **E.** Summary of HPV16 L2-specific neutralizing antibody titer analyzed with HPV16-SEAP pseudovirus based neutralization assay.

For convenience and improved stability, it is potentially beneficial to formulate a large batch of TA-CIN/GPI-0100 vaccine and aliquot it into single-use doses that can be frozen for long term storage. Because of its dodecyl group, GPI-0100 is more hydrophobic than its *Quillaja* saponin precursor and the avoidance of high ionic strength buffers and formulation in 5% (isotonic) mannitol is advantageous. Therefore, we formulated TA-CIN with GPI-0100 by mixing it overnight in 5% mannitol solution. Doses were frozen and stored at −80°C until needed for administration. To test whether a single freeze/thaw cycle impacts the immune response, we vaccinated naïve BALB/c mice with either freshly formulated TA-CIN/GPI-0100, or TA-CIN formulated with GPI-0100 in 5% mannitol and frozen to −80°C for storage until use. The mice were boosted twice as indicated in Fig. 2A. Two weeks after the last vaccination, splenocytes and sera were harvested to detect HPV16-specific T cells responses (Fig. 2B-D, S3A and C Fig.) and neutralizing antibody (Fig. 2E) respectively. Vaccination with TA-CIN/GPI-0100 induced a neutralizing antibody response against HPV16, whereas TA-CIN alone or GPI-0100 did not trigger a detectable (titer ≥50) neutralizing antibody response against HPV16 (Fig. 2E). With respect to cellular immunity, vaccination with TA-CIN alone was able to generate both TA-CIN-specific CD4^+^ and CD8^+^ T cell responses (Fig. 2B-D, S3A and C Fig.). However, formulating TA-CIN with GPI-0100 greatly enhanced both these T cell responses, notably the TA-CIN-specific CD4^+^ T cell responses by 7-fold (Fig. 2B, S3A and C Fig.). This CD4^+^ T cell response is at least in part L2-specific since HPV16 L2, but not mock transfected, CT26 cells were able to activate CD4^+^ T cells of vaccinated mice to produce IFN-γ (Fig. 2C, S3B Fig.). We also tested whether there is CD4+ T cell response against HPV16 E7 using a peptide that contains a previously reported CD4 T cell epitope [45] and found no detectable response (S4 Fig.). Importantly, mice vaccinated with TA-CIN formulated with GPI-0100 in mannitol solution and stored at −80°C generated similar TA-CIN-specific CD4^+^, CD8^+^ T cell and HPV16-specific neutralizing antibody responses to freshly formulated TA-CIN with GPI-0100 (Fig. 2). These data suggest that TA-CIN formulated with GPI-0100 in mannitol solution can be frozen and stored at −80°C for an extended period (potency was retained after 11 months storage, the longest time tested to date), without compromising its immunogenicity.

**Figure 3:**
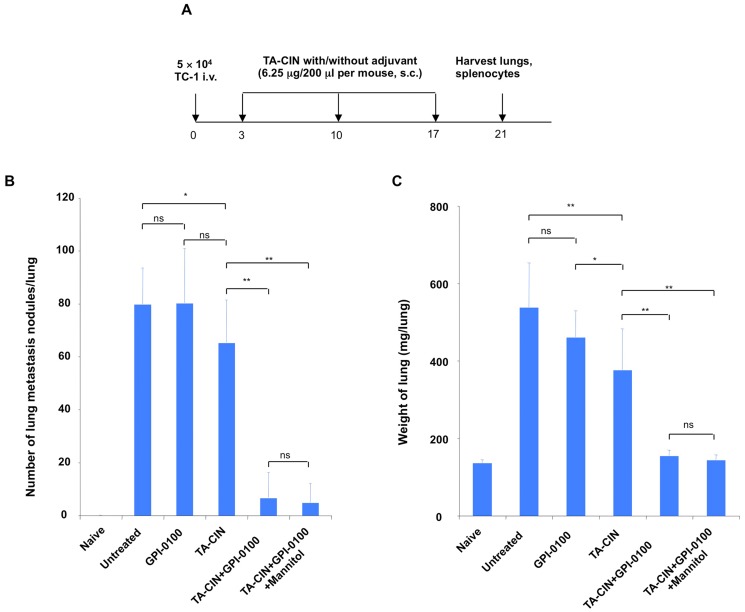
Anti-tumor to TA-CIN/GPI-0100 vaccination in the TC-1 lung metastasis model. **A.** Schematic illustration of the experimental protocol. Briefly, 5∼8 weeks old female C57BL/6 mice (10 mice/group) were injected with 5×10^4^ TC-1 cells intravenously on day 0. On day 3, the mice were vaccinated s.c. with either 6.25 µg/mouse of TA-CIN, or formulated with 50 µg of GPI-0100, or formulated with 50 µg of GPI-0100 in 50.7 mg/mL of mannitol and frozen/thawed once. The mice were boosted twice with the same regimen with 1-week interval. On day 21, the mice were sacrificed to harvest lungs and spleens. **B.** Summary of the number of TC-1 metastasis nodules (and photographs of lungs are presented in S5A Fig.). **C.** Summary of the weight of the lungs of the mice.

**Figure 4:**
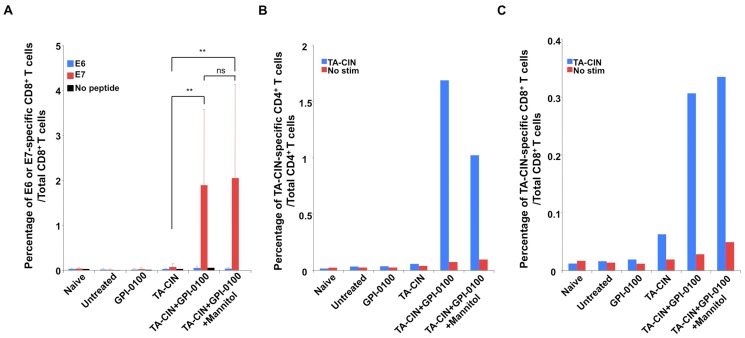
Anti-tumor and antigen-specific CD8^+^ T cell responses to TA-CIN/GPI-0100 vaccination in the TC-1 lung metastasis model. As described in Fig. 3A, 5∼8 weeks old female C57BL/6 mice (10 mice/group) were injected with 5×10^4^ TC-1 cells intravenously on day 0. On day 3, the mice were vaccinated s.c. with either 6.25 µg/mouse of TA-CIN, or formulated with 50 µg of GPI-0100, or formulated with 50 µg of GPI-0100 in 50.7 mg/mL of mannitol and frozen/thawed once. The mice were boosted twice with the same regimen with 1-week interval. On day 21, the mice were sacrificed to harvest lungs and spleens. **A.** Summary of flow cytometry analysis of HPV16 E6 and E7-specific CD8^+^ T cell responses analyzed by IFN-γ intracellular staining (and representative data is shown in S5B Fig.). **B.** Summary of the flow cytometry analysis of TA-CIN-specific CD4^+^ T cell responses analyzed by IFN-γ intracellular staining (and representative data is shown in S6A Fig.). **C.** Summary of the flow cytometry analysis of TA-CIN-specific CD8^+^ T cell responses analyzed by IFN-γ intracellular staining (and representative data is shown in S6B Fig.). The data were acquired with FACSCalibur and analyzed with CellQuest.

To further test whether the frozen formulation retains immunogenicity after a single freeze/thaw cycle, C57BL/6 mice bearing TC-1 tumor in the lungs were administered either TA-CIN freshly formulated with GPI-0100, or TA-CIN formulated with GPI-0100 in mannitol solution, stored at −80°C and thawed just prior to use. The mice were boosted twice as indicated in Fig. 3A, and 4 days after last vaccination, lungs and splenocytes were harvested to detect tumor growth and T cell responses. As shown in Fig. 3B, C and S5A Fig.), mice treated with GPI-0100 alone did not inhibit TC-1 tumor growth, and mice vaccinated with TA-CIN alone demonstrated a slight antitumor effect when compared to untreated mice. In contrast, mice vaccinated with TA-CIN/GPI-0100 greatly inhibited tumor growth when compared to TA-CIN or GPI-0100 alone treated mice, and the response was similar in the fresh and frozen/thawed formulations. Next we analyzed the CD8^+^ T cell responses against HPV16 E6/E7 elicited by vaccination (Fig. 4A and S5B Fig.). None of the groups exhibited significant E6-specific CD8^+^ T cell responses. TA-CIN vaccinated mice generated weak E7-specific CD8^+^ T cell responses (0.074±0.065% compared to 0.013±0.008% in untreated and 0.027±0.024% of GPI-0100 treated). In contrast, mice vaccinated with freshly formulated TA-CIN/GPI-0100 generated 25-fold more (1.888±1.679%, p = 0.003) and frozen formulation generated 27-fold more (2.049±2.078%, p = 0.008) E7-specific CD8^+^ T cells. E7-specific CD8^+^ T cells generated by the fresh or the frozen formulation of TA-CIN/GPI-0100 were comparable (p = 0.851).

Since a strong TA-CIN-specific CD4^+^ T cell response was observed after vaccination of naïve BALB/c mice with TA-CIN/GPI-0100 (Fig. 2B), we examined whether this TA-CIN-specific CD4^+^ T cell response was also induced in TC-1 tumor-bearing C57BL/6 mice after vaccination using both fresh and frozen vaccine. Both formulations of TA-CIN/GPI-0100 elicited robust TA-CIN-specific CD4^+^ T cell responses that were significantly greater than upon vaccination with TA-CIN alone (Fig. 4B and S5C Fig.). A similar pattern of response was observed for TA-CIN-specific CD8^+^ T cell responses (Fig. 4C and S5D Fig.). Taken together, our data from naïve BALB/c mice and TC-1 tumor-bearing C57BL/6 mice demonstrate that GPI-0100 is able to greatly enhance TA-CIN-specific CD4^+^, and E7-specific CD8^+^ T cell responses, and L2-specific neutralizing antibody titers in serum. Taken together, the data suggest that freezing of TA-CIN/GPI-0100 in the isotonic mannitol formulation did not reduce its immunogenicity.

### GPI-0100 enhances protective antibody response elicited by TA-CIN

**Figure 5:**
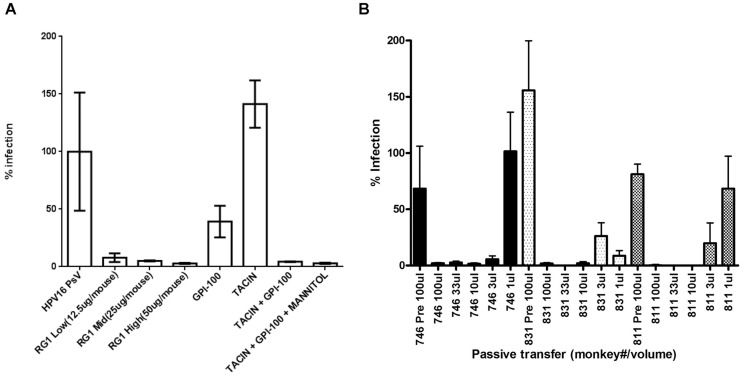
Passive transfer of TA-CIN antiserum protects against HPV pseudovirus infection of mouse vagina. A. Either purified L2-specific neutralizing monoclonal antibody (clone RG-1 at 12.5 25 or 50µg/mouse) or 100 µL pooled immune mouse serum (derived from the study shown in Fig. 2) was passively transferred i.p. into naïve mice. The immune serum used was pooled from groups of 5 mice previously vaccinated three times s.c. at 2 week intervals with either GPI-0100 alone, or 6.25 µg/mouse of TA-CIN alone, or formulated with 50 µg of GPI-0100, or formulated with 50 µg of GPI-0100 in 50.7 mg/mL of mannitol and frozen/thawed once (see schema in Fig. 2A). The mice were challenged intravaginally with HPV16 pseudovirions 24 h after the passive transfer and imaged 3 days later. B. Pre-immune sera (100 µL) or volumes between 1 µL and 100 µL of immune sera after three vaccinations with TA-CIN/GPI-0100 from 3 macaques (I.D 746, 811 or 831) collected in a prior study was passively transferred i.p. into groups of naïve mice (n = 5). The mice were challenged intravaginally with HPV58 pseudovirions 24 h later and imaged 72 hours thereafter to assess infectivity.

We previously demonstrated that formulation with GPI-0100 greatly enhances the HPV16 neutralizing antibody response in serum that is induced by TA-CIN vaccination [16], and that actively vaccinated mice are protected against cutaneous challenge with HPV16 pseudovirus (PsV). Since HPV16 causes cancer in mucosa lined organs, notably the female genital tract and oropharynx, we tested whether this enhanced antibody response can protect against vaginal mucosal challenge with HPV16 PsV. Prophylactic immunity induced by L2 vaccination is believed to be mediated by neutralizing antibodies; therefore, we determined the protective efficacy of passive transfer of immune sera into naïve mice prior to their challenge intra-vaginally with HPV16 PsV. We injected i.p. into groups of naïve Balb/c mice (n = 5) the immune sera pooled from the respective vaccinated mice groups of Fig. 2, or a titration of the L2-specific HPV16 neutralizing monoclonal antibody RG-1 as a positive control. Following passive transfer, these mice were then challenged intra-vaginally 24 hours later with HPV16 PsV carrying a luciferase reporter. At 72 hours after challenge, infectivity as assessed through luciferase reporter expression was examined by imaging and quantification of bioluminescence of the genital area. As shown in Fig. 5, passive transfer of sera from mice vaccinated with TA-CIN alone or GPI-0100 alone failed to protect naïve mice from HPV16 PsV infection. In contrast, passive transfer of sera of mice vaccinated with either freshly formulated or frozen TA-CIN/GPI-0100 formulations prevented HPV16 PsV infection of naïve mice. These data show that formulation of TA-CIN with GPI-0100 could greatly enhance HPV16-specific protective antibody response elicited by vaccination such that it protected even after passive transfer of sera at an effective dilution of ∼1∶20 in naïve mice. Formulation in mannitol and freezing did not reduce the ability of GPI-0100 to enhance the protective antibody response triggered by TA-CIN vaccination (Fig. 5A). Sera from GPI-0100 vaccinated mice neither showed any HPV16-specific neutralizing antibody titer when analyzed by *in vitro* assay nor a significant protective effect (Fig. 5A).

In our prior study [16], we showed that vaccination of three macaques with TA-CIN/GPI-0100 elicited broadly cross-neutralizing serum antibodies. To examine whether this response could provide cross-type protection, naïve Balb/c mice were also injected with pre-immune or a titration of the immune sera from these three macaques (I.D. 746, 811 and 831), and the mice were subsequently challenged intra-vaginally with HPV58 pseudovirions. As shown in Fig. 5B, the immune sera of the macaques strongly protected against HPV58 challenge and this effect could be titrated up to an effective dilution of ∼1∶600, whereas the pre-immunization serum sample was not protective at an effective dilution of ∼1∶20.

### Combination of chemotherapy and vaccination generated strong antitumor immunity

**Figure 6:**
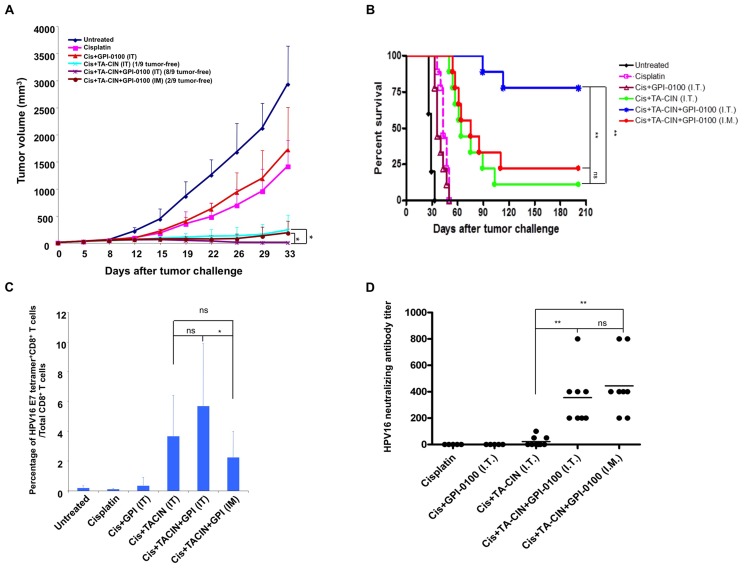
Effect of the combination of chemotherapy and TA-CIN vaccination on the anti-tumor effect, HPV16 E7-specific CD8+ T cell and L2-specific neutralizing antibody responses in TC-1 tumor bearing mice. **A.** Briefly, 5∼8 weeks old C57BL/6 mice (5–9 mice per group) were injected with 1×10^5^ of TC-1 tumor cells subcutaneously. The tumor-bearing mice were divided into 6 groups; one untreated, and 5 other groups were given 5 mg/kg of cisplatin (Cis) via intraperitoneal injection on day 5 and 12. Of the 5 groups that were treated with cisplatin, one was not vaccinated (Cisplatin) and another received intratumoral GPI-0100 alone (Cis+GPI-0100 IT), another was vaccinated intratumorally with 6.25 µg/mouse TA-CIN alone in 20 µL (Cis+TA-CIN IT), and the last two groups were given 6.25 µg/mouse TA-CIN formulated with 50 µg of GPI-0100 in 20 µL mannitol solution (frozen/thawed once) via intramuscular injection (Cis+TA-CIN+GPI-0100 I.M.) or via intratumor injection (Cis+TA-CIN+GPI-0100 I.T.). In all groups, the vaccinations were given on days 6, 13 and 20. An illustrative schema is shown in S7 Fig. **A.** Summary of the TC-1 tumor growth volume. **B.** Kaplan and Meier survival analysis of TC-1 tumor cell challenged mice treated with the different regimens. **C.** Summary of flow cytometry analysis of HPV16 E7-specific CD8^+^ T cell responses analyzed by HPV16 E7 tetramer staining. The data were acquired with FACSCalibur and analyzed with CellQuest (and representative data is shown in S8 Fig.). **D.** Summary of HPV16 neutralizing antibody titer analyzed with HPV16-SEAP pseudovirus based neutralization assay.

Vaccination with TA-CIN/GPI-0100 reduced but did not eliminate metastatic TC-1 tumors (Figs. 1 and 3), suggesting that this immunotherapy might be more effective if combined with other conventional approaches for therapy of HPV-associated cancers, such as treatment with the chemotherapeutic agent cisplatin. Recently, we demonstrated that treatment of TC-1 tumor-bearing mice with cisplatin transiently converted the tumor microenvironment into a highly permissive state for intra-tumor vaccination resulting in dramatically improved antitumor immunity [35]. After cisplatin treatment, vaccination with TA-CIN alone via intra-tumoral (i.t.) administration induced strong HPV16 E7-specific CD8^+^ T cell responses and resulted in the complete eradication of the established tumors, although in this study a high amount (50 µg) of TA-CIN and intensive administration schedule (3 vaccinations in 6 days) was used. We hypothesized that formulation with GPI-0100 might provide a dose sparing effect via enhancing the antitumor immune response to vaccination after cisplatin therapy with lower doses of TA-CIN. To address this question we treated C57BL/6 mice bearing a subcutaneous TC-1 tumor as illustrated in S7 Fig. using 6.25 µg TA-CIN dose for each vaccination, and GPI-0100 formulated TA-CIN from a frozen preparation. Intra-tumor vaccination with TA-CIN or TA-CIN/GPI-0100 significantly inhibited TC-1 tumor growth compared to cisplatin or cisplatin plus GPI-0100 treatment (Fig. 6A). On day 33 post TC-1 tumor cell challenge, only 1 out of 9 mice vaccinated i.t. with TA-CIN alone was tumor free. In contrast, 8 of 9 mice that received i.t. vaccination with TA-CIN/GPI-0100 were tumor free. After cisplatin treatment, intramuscular vaccination with TA-CIN/GPI-0100 also inhibited TC-1 tumor growth significantly such that 2 out of 9 mice were tumor free on day 33 post tumor challenge. Furthermore, vaccination of mice i.t. with TA-CIN alone, or TA-CIN/GPI-0100 via either i.t. or intramuscular injection significantly prolonged the survival of the tumor-bearing mice (Fig. 6B). However, i.t. vaccination with TA-CIN/GPI-0100 after cisplatin treatment was most efficacious and the response was durable>200 days (Fig. 6A and B). These data suggest that formulation with GPI-0100 is able to decrease the dosage (from 50 µg to 6.25 µg) of TA-CIN needed for durable tumor rejection after i.t. injection of cisplatin-treated mice. Intramuscular vaccination with TA-CIN/GPI-0100 after cisplatin treatment elicited an anti-tumor effect comparable to i.t. vaccination with TA-CIN alone.

To analyze the E7-specific CD8+ T cell responses to vaccination of the tumor bearing mice in the above study, we stained CD8^+^ T cells in the peripheral blood with HPV16 E7aa49-57 peptide loaded H-2D^b^ tetramer (Fig. 6C and S8 Fig.). Consistent with our previous data [35], i.t. vaccination with TA-CIN alone after cisplatin treatment elicited a strong E7-specific CD8^+^ T cell response. Addition of GPI-0100 did not significantly increase the E7-specific CD8^+^ T cell response detected in peripheral blood, suggesting that the cisplatin treatment primed or served as an adjuvant to TA-CIN vaccination in the tumor bearing mice. However, no detectable response was seen after either cisplatin treatment alone or with i.t. administration of GPI-0100 alone. Intramuscular vaccination with TA-CIN/GPI-0100 also generated a similarly robust systemic E7-specific CD8^+^ T cell response as detected in the peripheral blood. Finally, the HPV16-specific neutralizing antibody response was measured in sera of TC-1 tumor-bearing mice after treatment (Fig. 6D). After cisplatin treatment of tumor-bearing mice, the generation of a robust HPV16-specific neutralizing antibody response was still dependent on the formulation of TA-CIN with GPI-0100 and was similar whether the TA-CIN/GPI-0100 was administered into muscle or directly into the tumor.

## Discussion

Our findings build upon earlier studies supporting both the potential preventive and therapeutic efficacy of the TA-CIN vaccine, especially when formulated with the GPI-0100 adjuvant. Vaccination of mice with TA-CIN elicits a neutralizing antibody response that is protective against both skin [16] and intra-vaginal challenge with high doses of HPV16 pseudovirions. This protection is likely mediated by neutralization of the viral inoculum since passive transfer of the immune serum is sufficient to confer immunity.

In early phase clinical trials, vaccination of healthy volunteers and high grade vulvar intraepithelial neoplasia (VIN) patients three times with up to 500 µg of TA-CIN was well tolerated, and induced HPV-specific antibody and T cell responses [17], although only weak neutralizing antibody titers against HPV16 and HPV18 of uncertain duration were noted. Furthermore, especially in VIN/VaIN patients, the titers were low and responses inconsistent [46], suggesting the need for a potent adjuvant. We previously demonstrated that vaccination three times with 125 µg of TA-CIN formulated with 1 mg GPI-0100 (corresponding to 20-fold the TA-CIN/GPI-0100 dose used in the murine studies herein) was well tolerated by 3 macaques and elicited robust HPV neutralizing and cross-neutralizing antibodies in each [16]. Whereas L1 VLP immunity is type restricted, L2 elicits broadly cross-type reactive responses because of the sequence conservation of its linear protective epitopes. For example, vaccination with HPV16 L1 VLP does not protect against HPV58, although both are members of the α9 species and have a high degree of sequence homology. Here we show that passive transfer of the macaque-derived TA-CIN/GPI-0100 antisera protected mice from vaginal challenge with HPV58, a non-vaccine type, with titers greater than those previously observed with sera of patients vaccinated with TA-CIN alone [46]. Thus TA-CIN/GPI-0100 vaccination might protect against future infection (with a different type or HPV16 after its clearance) or potentially limit spread of infection within the host [47]. However, with the advent of highly multivalent L1 VLP vaccines (e.g. Gardasil 9), the main major unmet medical need remains a therapeutic HPV vaccine.

Vaccination with L2 has been shown to clear an L2-expressing tumor [12] or papillomas [13], and therefore might contribute to immune clearance of productive lesions. Indeed, we show that vaccination with TA-CIN/GPI-0100 elicits a robust CD4 T cell proliferative response in mice, and proliferative responses to TA-CIN are also observed in patients. However, while L2 is expressed in precursor lesions, it is not detectable in invasive cervical cancers [11].

We previously also showed that vaccination with TA-CIN/GPI-0100 elicits robust E6/E7-specific CD8 T cell immune responses and protects mice from experimental challenge with the TC-1 tumor model. Since TC-1 cells do not have the L2 gene and both E6 and E7 are intracellular, this implies that protection occurs via E6/E7-specific cell-mediated immunity. Although it is not clear if protection against TC-1 challenge translates to protection against viral challenge, studies in rabbits have shown that vaccination against E6 and E7 prevents warts after CRPV inoculation [48], [49]. Thus TA-CIN/GPI-0100 vaccination may provide prophylaxis against HPV16 infection by both L2-specific antibody and E6/E7-specific T cell immunity.

The need for a therapeutic HPV vaccine, especially for patients with HPV-associated cancer, is pressing. Prophylactic vaccination with L1 VLP is recommended for younger patients, but does not treat existing disease that is prevalent in older populations, and its cost is borne in addition to cytologic screening. Prophylaxis aims for vaccination of the entire at risk population, whereas therapeutic vaccination could potentially only be administered to persistently infected patients identified by routine HPV DNA testing. This may be particularly relevant for patients with persistent oral HPV16 infections who are potentially at risk for the development of HPV16+ HNSCC, a disease for which no cytologic screening currently existing. This approach also has potential for the treatment of persistent HPV16 infection associated with <CIN2, rather than continued close follow-up until clearance or progression to CIN2+ and therapeutic loop electrosurgical excision procedure (LEEP). While LEEP is effective, it is associated with significant side effects (such as cervical incompetence and preterm delivery [50]), suggesting that vaccination might represent an alternative approach. Although ablative treatment has proven effective for CIN2/3, it is often less effective for high grade VIN/VaIN/AIN and associated with significant morbidity [51]. Immunotherapy of VIN/VaIN with the combination of topical imiquimod followed by systemic TA-CIN vaccination has shown promise in this regard [17]. Clearance of disease was associated with a greater systemic T cell proliferative response for TA-CIN [17], and in our preclinical studies, the formulation of TA-CIN with GPI-0100 profoundly enhanced the systemic T cell proliferative response for TA-CIN in mice.

Larger and more advanced HPV-related disease has been associated with greater immune suppression within the lesional microenvironment that has proven a major barrier to immunotherapies [52]. Several well-established cancer treatments, such as radiation and chemotherapy, not only directly kill tumor cells and shrink the lesion via DNA damage, but they also elicit an immunogenic tumor cell death and enhance antitumor immunity [53], [54]. In preclinical studies, prior cisplatin therapy increased MHC class I expression by the TC-1 tumor cells, rending them more susceptible to killing by E7-specific cytotoxic T cells, and was associated with reductions in the myeloid suppressor cell and T regulatory cell populations in the spleen. Cisplatin therapy also enhanced the recruitment of cytotoxic T cells to the tumor site and enhanced their proliferation locally, likely via antigen release from dying tumor cells. Here we show that local vaccination with TA-CIN at the tumor site after cisplatin administration enhanced the systemic E7-specific cytotoxic T cell response, as previously observed after local radiation therapy [55]. This may reflect the accumulation of CD11c+ dendritic cells in tumor loci after cisplatin therapy available take up and present peptides derived from the locally injected TA-CIN [34], [55]. Recruitment of CD11c+ dendritic cells has been shown to occur upon the release of damage-associated molecular patterns (DAMPs) by dying cancer cells, and can trigger antigen uptake, maturation and presentation, via recognition by the TLR4 and activation of the type I interferon pathway. The presence of the DAMPs may adjuvant the injected TA-CIN, potentially accounting for the benefit of local over remote i.m. immunization after cisplatin therapy. Consistent with this hypothesis, we recently observed that intratumoral injection of E7 peptide after irradiation of TC-1 tumor bearing TLR4−/− or IFNAR−/− mice elicited weaker E7-specific cellular immune responses than wild type mice[55].

Topical administration of the TLR7 agonist imiquimod elicits a potent type I interferon response and has efficacy for the treatment of genital warts, VIN and CIN, presumably by enhancing and targeting spontaneous immunity and counteracting a suppressive microenvironment [17], [56], [57]. Notably, intra-tumoral vaccination with TA-CIN/GPI-0100 was more effective that local vaccination with TA-CIN alone in cisplatin-treated mice with subcutaneous TC-1 tumor, although the immune pathways activated by GPI-0100 adjuvant have yet to be defined. This suggests that the GPI-0100 might alter the locally suppressive tumor microenvironment, which is a likely factor in the failure of therapeutic vaccination in bulkier disease. A variety of immunosuppressive mechanisms have been associated with poorer outcomes in cancer patients; these include elevated numbers of regulatory T cells, CD11b+ myeloid-derived suppressor cells (MDSC), CD11b+ F4/80+ tumor-associated macrophages (TAMs), and their local induction of PD-L1, IDO, arginase [52]. Local radiation therapy has been shown to recruit both MDSC and TAMs to the tumor bed which might potentially counteract the local immune response. Again, we recently demonstrated that CD11b+ myeloid cells (but not TAM) derived from the tumor of irradiated mice will take up and present locally injected E7 peptide, rendering them susceptible to killing by E7-specific T cells [55]. A similar mechanism may also contribute to the improved response to local immunization with TA-CIN after cisplatin therapy, as compared with remote i.m. injection.

Intratumoral vaccination is technically more challenging and less practical than intramuscular vaccination. As intramuscular vaccination with TA-CIN/GPI-0100 produced an immunotherapeutic response against TC1 tumor in cisplatin-treated mice of similar efficacy to intratumoral injection of TA-CIN alone, this suggests that formulation with GPI-0100 may overcome the need for intratumoral vaccination of TA-CIN, although the latter is optimal. Another issue for local vaccination with TA-CIN is whether each tumor site must be injected in hosts bearing disseminated lesions. This remains to be determined, but i.m. vaccination with TA-CIN/GPI-0100 was effective in the TC-1 tumor model of hematogeneous metastasis to the lungs.

The TA-CIN vaccine contains both E6 and E7. E7 is clearly the immunodominant antigen in the C57BL6 mouse, but in humans several studies suggest that E6 may be dominant in the majority of patients, although E7 is immunogenic. Furthermore, a broader response is associated with improved clinical outcome after vaccination, further suggesting the merit of targeting both viral oncoproteins in HPV16+ patients.

## Supporting Information

S1 Fig **Comparison of the immunogenicity of TA-CIN formulated with GPI-0100 and administered by either intramuscular (i.m.) or subcutaneous (s.c.) injection.** **A**. Schematic illustration of the experiment. Briefly, 5∼8 weeks old female C57BL/6 mice (5 mice/group) were vaccinated with 25 µg/mouse of TA-CIN formulated with 50 µg of GPI-0100 by either i.m. or s.c. injection. The mice were boosted twice with the same regimen with 2-week intervals. One week after the last vaccination, sera and splenocytes were harvested. **B**. Flow cytometry analysis of representative E6 and E7-specific CD8^+^ T cell response in splenocytes by intracellular IFN-γ staining. **C**. Summary of HPV16 neutralizing antibody titer determined *in vitro* by HPV16-SEAP pseudovirus based neutralization assay.
(TIF)

S2 Fig **Image of lungs and representative HPV16 E6 and E7-specific CD8^+^ T cell responses induced by TA-CIN/GPI-0100 vaccination of mice with hematogenously disseminated TC-1 tumor.** The experiment was performed as illustrated in Fig. 1A. On day 27 after TC-1 tumor cell injection, the mice were sacrificed to harvest lungs and spleens. **A**. Image of TC-1 tumor lung nodules (summarized in Fig. 1B). **B**. Representative of flow cytometry images of HPV16 E6 and E7-specific CD8^+^ T cell responses analyzed by IFN-γ intracellular staining. The data were acquired with FACSCalibur and analyzed with CellQuest (summarized in Fig. 1D).
(TIF)

S3 Fig **Representative of flow cytometry images of Effect of freezing on TA-CIN-specific and HPV16 L2-specific T cell responses induced by GPI-0100 formulated TA-CIN vaccination.** The experiment was performed as illustrated in Fig. 2A. Two weeks after the last vaccination, splenocytes were harvested and stimulated with either TA-CIN or CT26 cells transfected with HPV16 L2. **A**. Representative of flow cytometry images of TA-CIN-specific CD4^+^ T cell responses analyzed by IFN-γ intracellular staining (summarized in Fig. 2B). **B**. Representative of flow cytometry images demonstrating that HPV16 L2-specific CD4^+^ T cell responses induced by TA-CIN formulated with GPI-0100 in mannitol and frozen/thawed once (summarized in Fig. 2C). **C**. Representative of flow cytometry images of TA-CIN-specific CD8^+^ T cell responses analyzed by IFN-γ intracellular staining (summarized in Fig. 2D).
(TIF)

S4 Fig **Analysis of HPV16 E7-specific CD4^+^ T cell responses induced by GPI-0100 formulated TA-CIN.** **A**. Schematic illustration of the experimental protocol. Briefly, 5∼8 weeks old female C57BL/6 mice (5 mice/group) were vaccinated subcutaneously with 25 µg/mouse of TA-CIN formulated with 50 µg of GPI-0100. The mice were boosted twice with the same regimen with 2-week interval. One week after the last vaccination, splenocytes were harvested and stimulated with HPV16 E7aa30-67 peptide (10 µg/ml) at the presence of GoligiPlug at 37°C overnight. **B**. Representative of flow cytometric analysis of HPV16 E7-specific CD4^+^ T cell responses analyzed by IFN-γ intracellular staining. **C**. Summary of the flow cytometry analysis.
(TIF)

S5 Fig **Image of lung nodules and representative of flow cytometry images of HPV16 E6 and E7-specific T cell responses after TA-CIN/GPI-0100 vaccination in the TC-1 lung metastasis model.** The experiment was performed as illustrated in Fig. 3A. On day 21 after TC-1 tumor cell injection, the mice were sacrificed to harvest lungs and spleens. **A**. Image of TC-1 lung metastasis nodules (summarized in Fig. 3B). **B**. Representative of flow cytometry images of HPV16 E6 and E7-specific CD8^+^ T cell responses analyzed by IFN-γ intracellular staining (summarized in Fig. 3D). **C**. Representative of flow cytometry images of TA-CIN-specific CD4^+^ T cell responses analyzed by IFN-γ intracellular staining (summarized in Fig. 3E). **D**. Representative of flow cytometry images of TA-CIN-specific CD8^+^ T cell responses analyzed by IFN-γ intracellular staining (summarized in Fig. 3F).
(TIF)

S6 Fig **Representative of flow cytometry images of TA-CIN-specific T cell responses induced by TA-CIN/GPI-0100 vaccination in the TC-1 lung metastasis model.** The experiment was performed as illustrated in Fig. 3A. On day 21 after TC-1 tumor cell injection, the mice were sacrificed to harvest lungs and spleens. **A**. Representative of flow cytometry images of TA-CIN-specific CD4^+^ T cell responses analyzed by IFN-γ intracellular staining (summarized in Fig. 3E). **B**. Representative of flow cytometry images of TA-CIN-specific CD8^+^ T cell responses analyzed by IFN-γ intracellular staining (summarized in Fig. 3F).
(TIF)

S7 Fig **Schema of experiment testing the therapeutic efficacy of combined chemotherapy and TA-CIN vaccination of TC-1 tumor bearing mice.** **A**. Schematic illustration of the experimental protocol.
(TIF)

S8 Fig **Representative of flow cytometry images of HPV16 E7-specific CD8^+^ T cell responses after chemotherapy and TA-CIN vaccination in TC-1 tumor bearing mice.** The experiment was performed as illustrated in S7 Fig. One week after the last vaccination, PBMCs were prepared from the tumor-bearing mice, and stained with FITC-conjugated anti-mouse CD8a and PE-Conjugated HPV16 E7aa49-57 peptide loaded H-2D^b^ tetramer. The data were acquired with FACSCalibur and analyzed with CellQuest (summarized in Fig. 6C).
(TIF)
