# Supplementary figures and images for: Sequential Cisplatin Therapy and Vaccination with HPV16 E6E7L2 Fusion Protein in Saponin Adjuvant GPI-0100 for the Treatment of a Model HPV16+ Cancer
Source: PLoS One. 2015 Jan 5;10(1):e116389. doi: 10.1371/journal.pone.0116389 (PMC4283968; doi:10.1371/journal.pone.0116389)

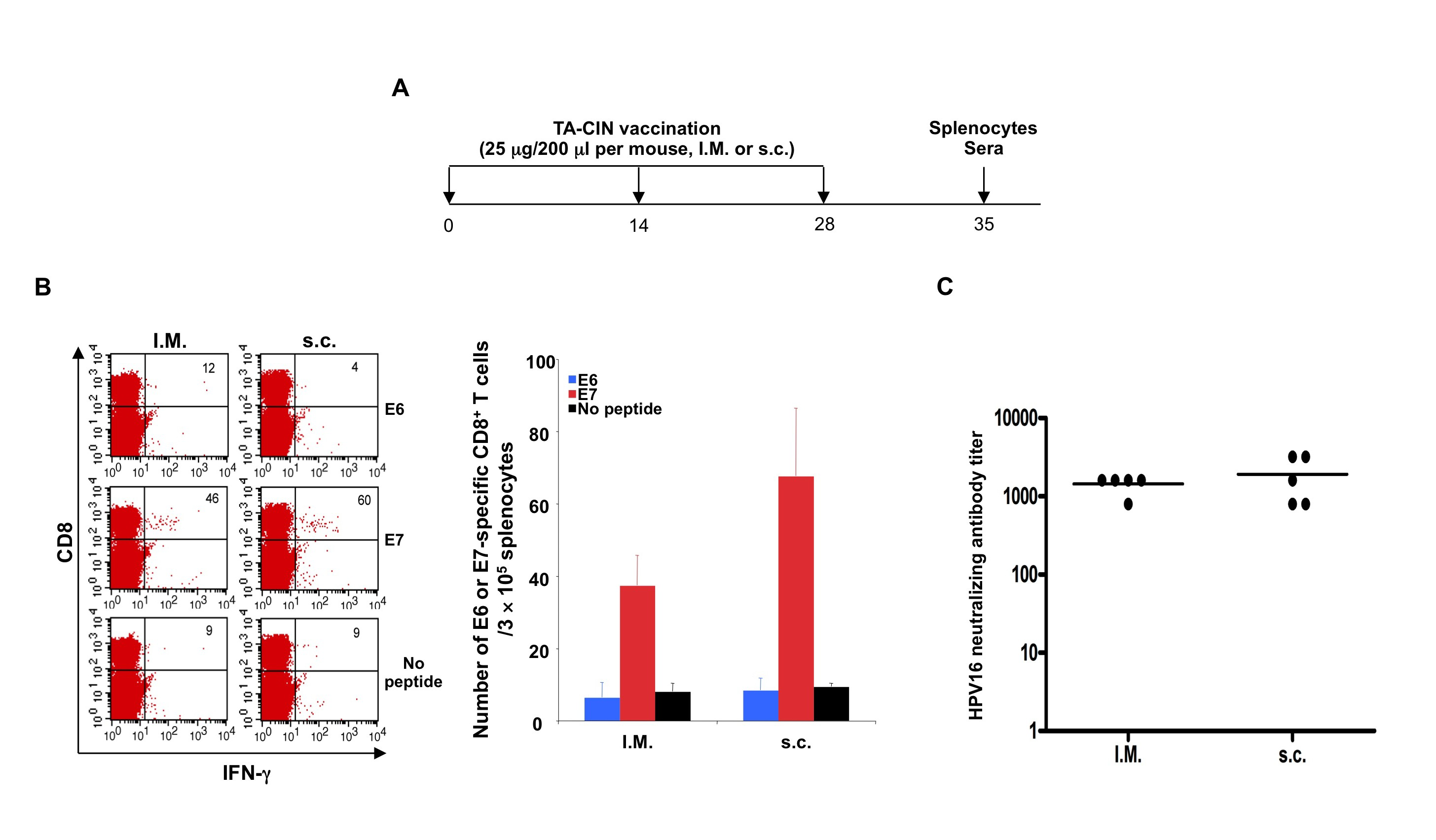

Supplement: S1 Fig — Comparison of the immunogenicity of TA-CIN formulated with GPI-0100 and administered by either intramuscular (i.m.) or subcutaneous (s.c.) injection. A. Schematic illustration of the experiment. Briefly, 5∼8 weeks old female C57BL/6 mice (5 mice/group) were vaccinated with 25 µg/mouse of TA-CIN formulated with 50 µg of GPI-0100 by either i.m. or s.c. injection. The mice were boosted twice with the same regimen with 2-week intervals. One week after the last vaccination, sera and splenocytes were harvested. B. Flow cytometry analysis of representative E6 and E7-specific CD8+ T cell response in splenocytes by intracellular IFN-γ staining. C. Summary of HPV16 neutralizing antibody titer determined in vitro by HPV16-SEAP pseudovirus based neutralization assay. (TIF) [file pone.0116389.s001.tif]

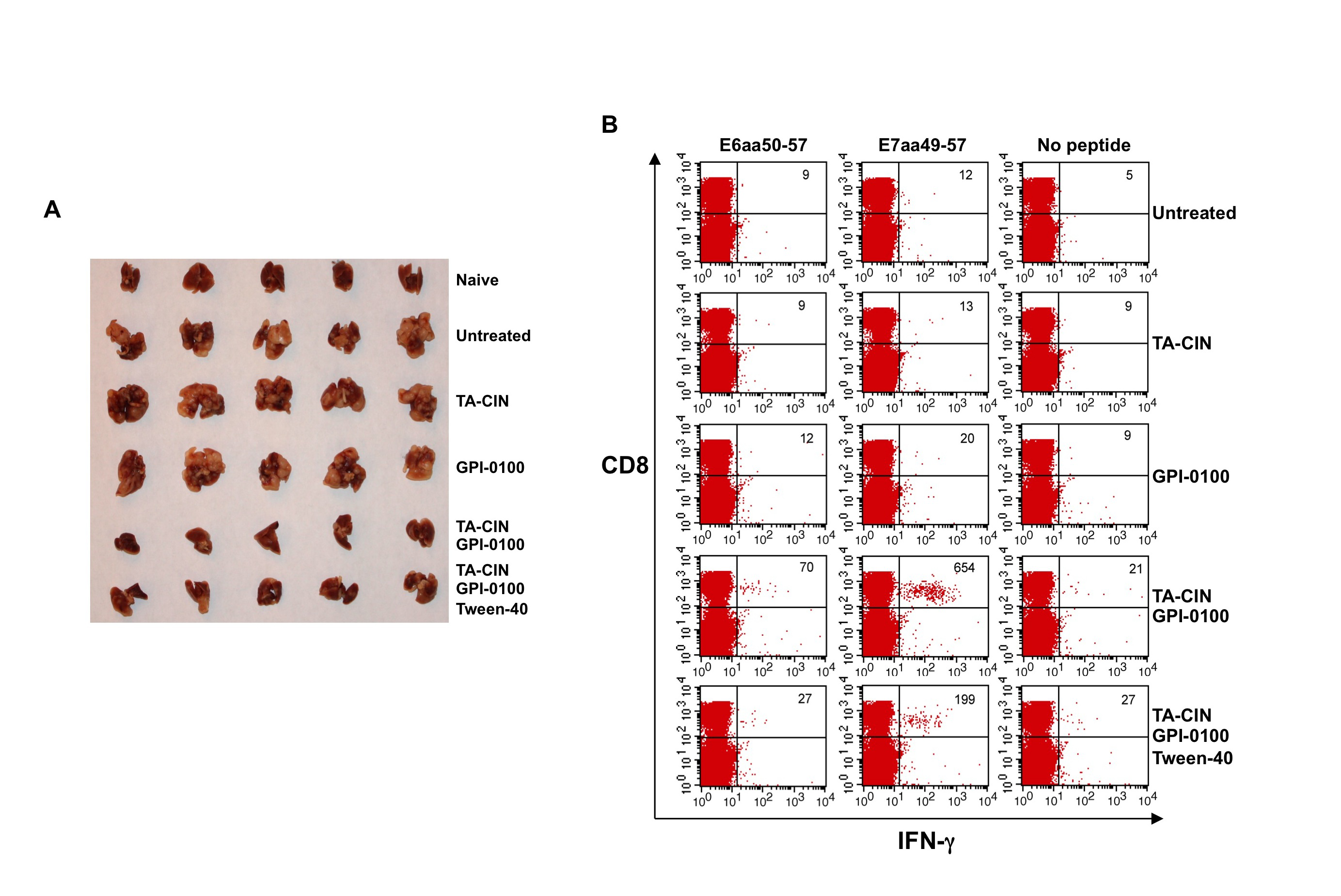

Supplement: S2 Fig — Image of lungs and representative HPV16 E6 and E7-specific CD8+ T cell responses induced by TA-CIN/GPI-0100 vaccination of mice with hematogenously disseminated TC-1 tumor. The experiment was performed as illustrated in Fig. 1A. On day 27 after TC-1 tumor cell injection, the mice were sacrificed to harvest lungs and spleens. A. Image of TC-1 tumor lung nodules (summarized in Fig. 1B). B. Representative of flow cytometry images of HPV16 E6 and E7-specific CD8+ T cell responses analyzed by IFN-γ intracellular staining. The data were acquired with FACSCalibur and analyzed with CellQuest (summarized in Fig. 1D). (TIF) [file pone.0116389.s002.tif]

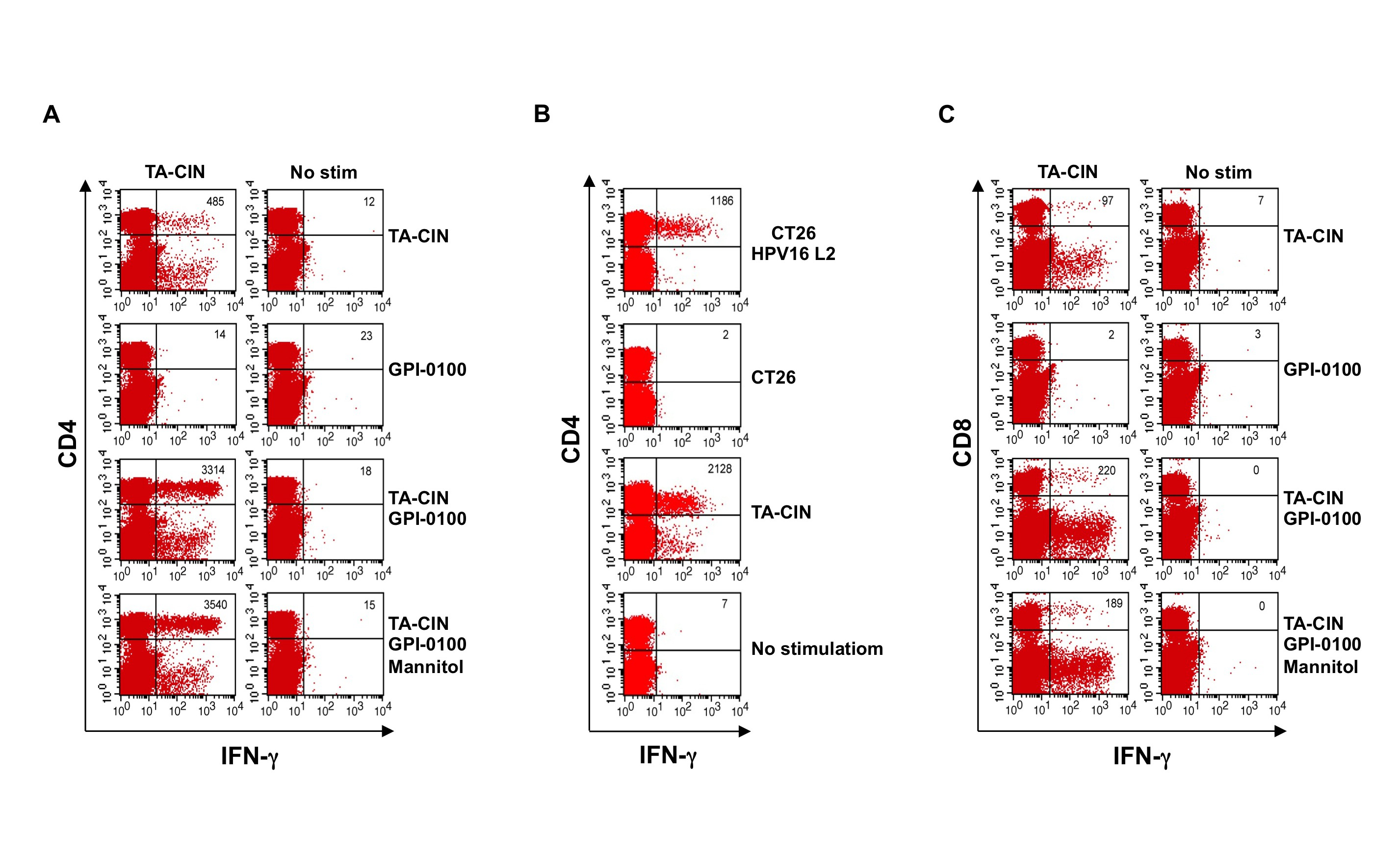

Supplement: S3 Fig — Representative of flow cytometry images of Effect of freezing on TA-CIN-specific and HPV16 L2-specific T cell responses induced by GPI-0100 formulated TA-CIN vaccination. The experiment was performed as illustrated in Fig. 2A. Two weeks after the last vaccination, splenocytes were harvested and stimulated with either TA-CIN or CT26 cells transfected with HPV16 L2. A. Representative of flow cytometry images of TA-CIN-specific CD4+ T cell responses analyzed by IFN-γ intracellular staining (summarized in Fig. 2B). B. Representative of flow cytometry images demonstrating that HPV16 L2-specific CD4+ T cell responses induced by TA-CIN formulated with GPI-0100 in mannitol and frozen/thawed once (summarized in Fig. 2C). C. Representative of flow cytometry images of TA-CIN-specific CD8+ T cell responses analyzed by IFN-γ intracellular staining (summarized in Fig. 2D). (TIF) [file pone.0116389.s003.tif]

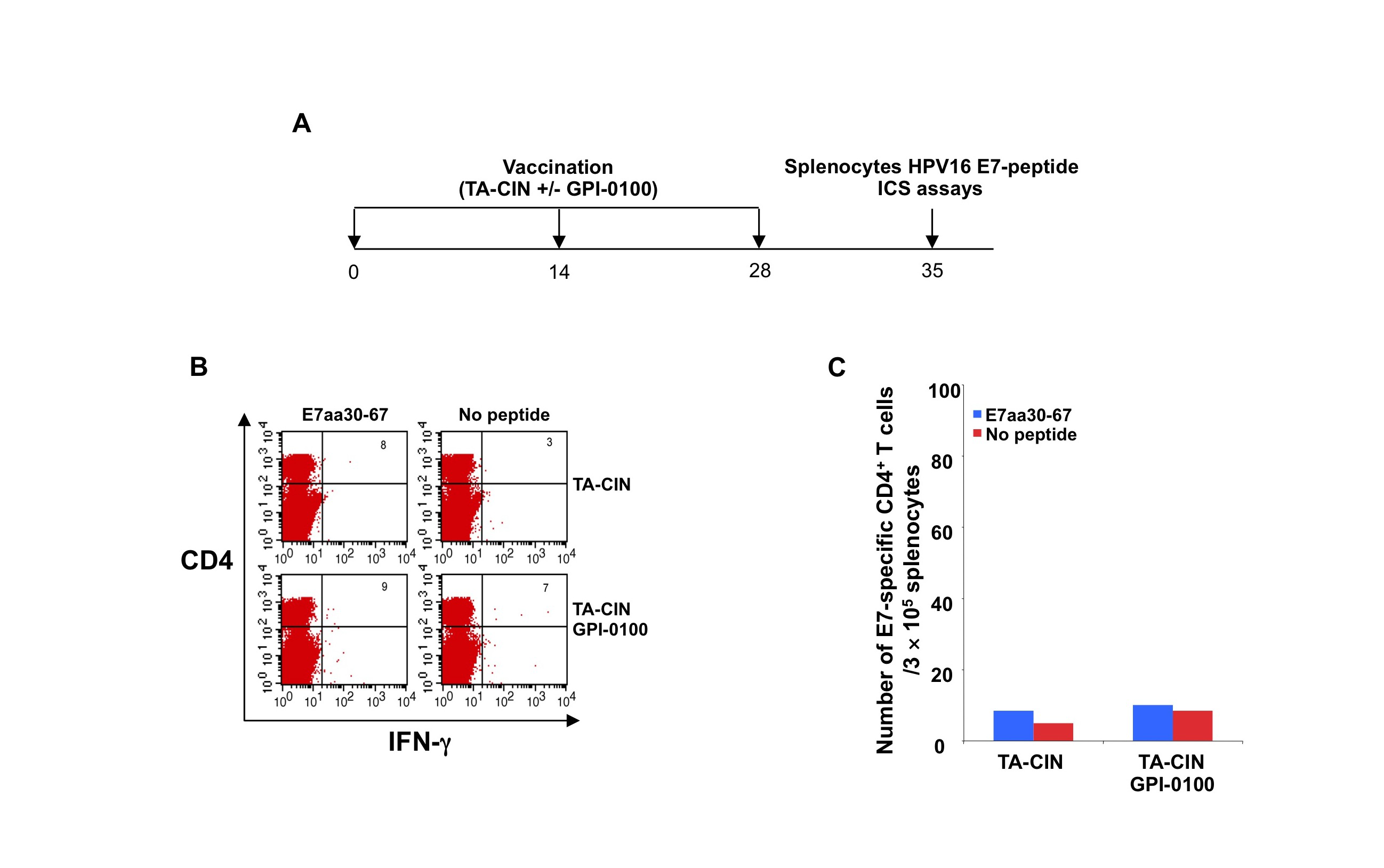

Supplement: S4 Fig — Analysis of HPV16 E7-specific CD4+ T cell responses induced by GPI-0100 formulated TA-CIN. A. Schematic illustration of the experimental protocol. Briefly, 5∼8 weeks old female C57BL/6 mice (5 mice/group) were vaccinated subcutaneously with 25 µg/mouse of TA-CIN formulated with 50 µg of GPI-0100. The mice were boosted twice with the same regimen with 2-week interval. One week after the last vaccination, splenocytes were harvested and stimulated with HPV16 E7aa30-67 peptide (10 µg/ml) at the presence of GoligiPlug at 37°C overnight. B. Representative of flow cytometric analysis of HPV16 E7-specific CD4+ T cell responses analyzed by IFN-γ intracellular staining. C. Summary of the flow cytometry analysis. (TIF) [file pone.0116389.s004.tif]

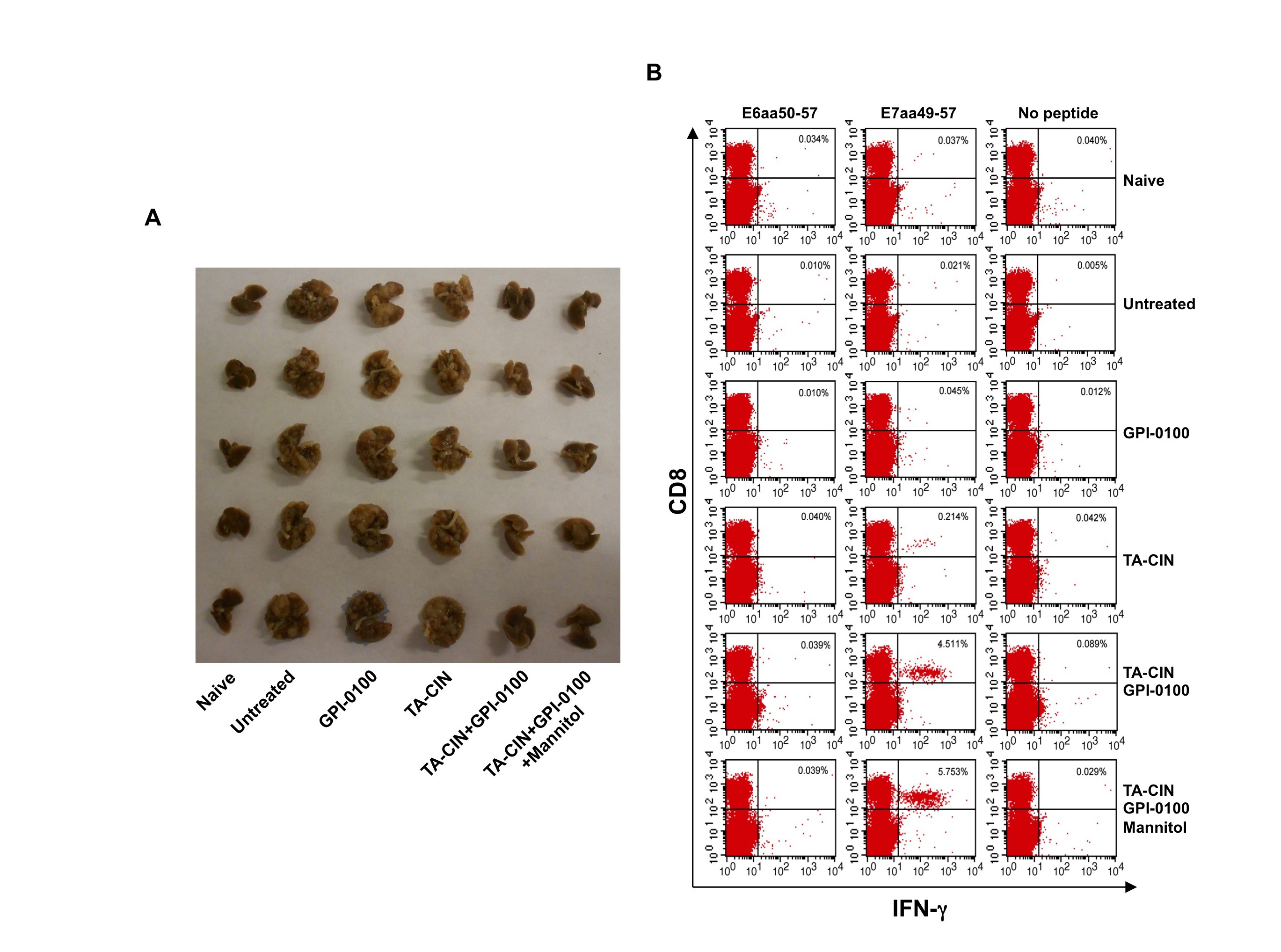

Supplement: S5 Fig — Image of lung nodules and representative of flow cytometry images of HPV16 E6 and E7-specific T cell responses after TA-CIN/GPI-0100 vaccination in the TC-1 lung metastasis model. The experiment was performed as illustrated in Fig. 3A. On day 21 after TC-1 tumor cell injection, the mice were sacrificed to harvest lungs and spleens. A. Image of TC-1 lung metastasis nodules (summarized in Fig. 3B). B. Representative of flow cytometry images of HPV16 E6 and E7-specific CD8+ T cell responses analyzed by IFN-γ intracellular staining (summarized in Fig. 3D). C. Representative of flow cytometry images of TA-CIN-specific CD4+ T cell responses analyzed by IFN-γ intracellular staining (summarized in Fig. 3E). D. Representative of flow cytometry images of TA-CIN-specific CD8+ T cell responses analyzed by IFN-γ intracellular staining (summarized in Fig. 3F). (TIF) [file pone.0116389.s005.tif]

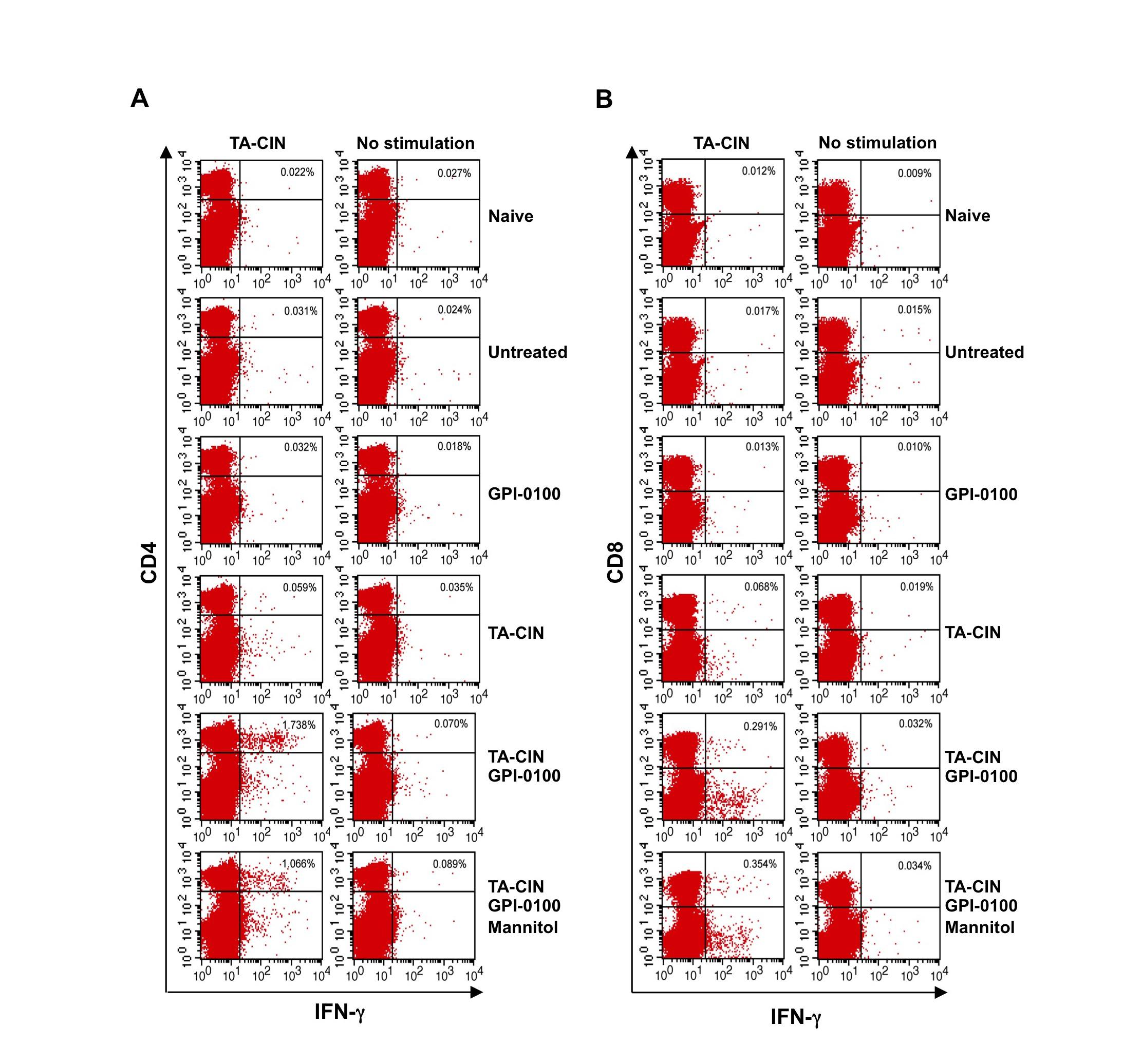

Supplement: S6 Fig — Representative of flow cytometry images of TA-CIN-specific T cell responses induced by TA-CIN/GPI-0100 vaccination in the TC-1 lung metastasis model. The experiment was performed as illustrated in Fig. 3A. On day 21 after TC-1 tumor cell injection, the mice were sacrificed to harvest lungs and spleens. A. Representative of flow cytometry images of TA-CIN-specific CD4+ T cell responses analyzed by IFN-γ intracellular staining (summarized in Fig. 3E). B. Representative of flow cytometry images of TA-CIN-specific CD8+ T cell responses analyzed by IFN-γ intracellular staining (summarized in Fig. 3F). (TIF) [file pone.0116389.s006.tif]

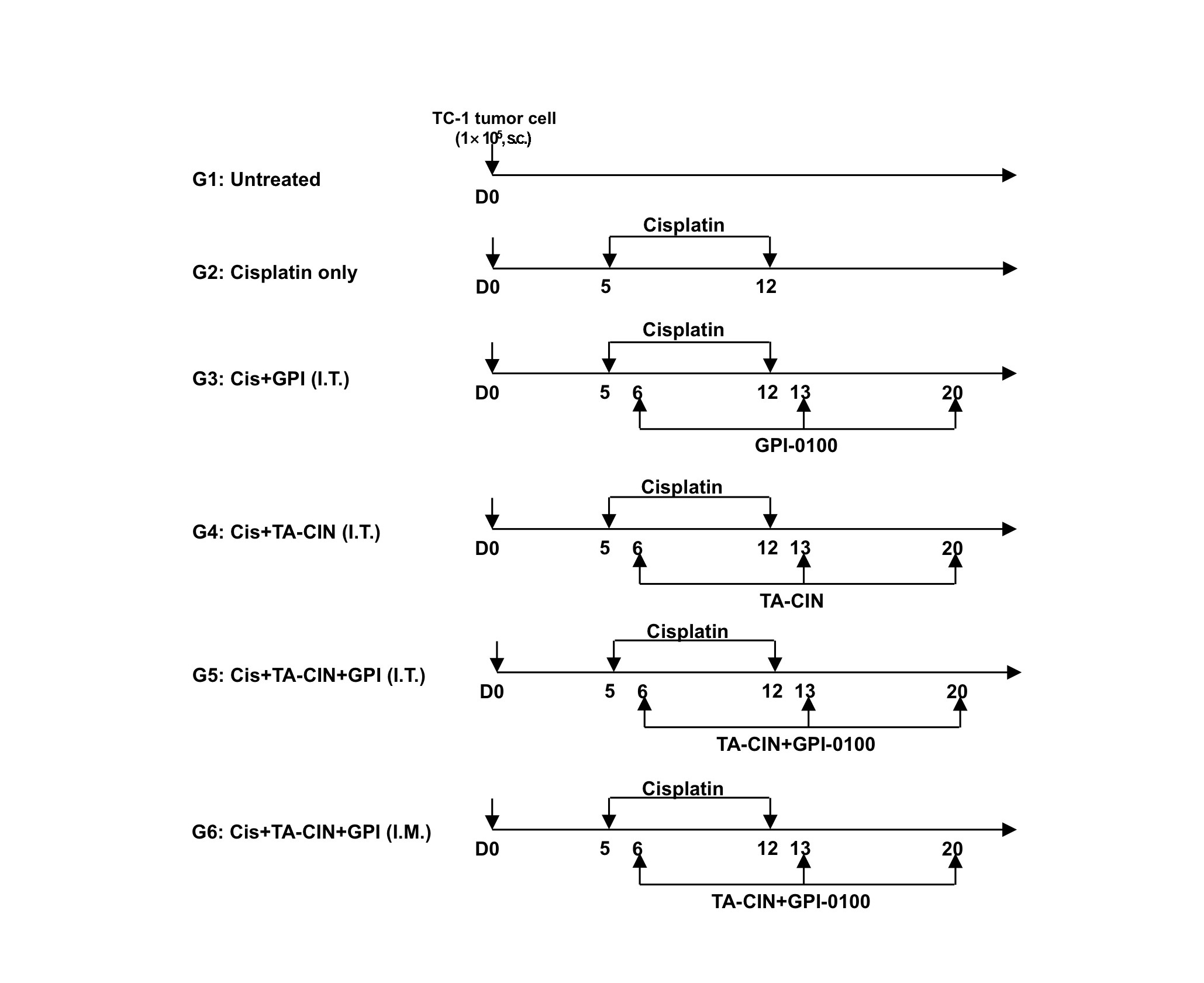

Supplement: S7 Fig — Schema of experiment testing the therapeutic efficacy of combined chemotherapy and TA-CIN vaccination of TC-1 tumor bearing mice. A. Schematic illustration of the experimental protocol. (TIF) [file pone.0116389.s007.tif]

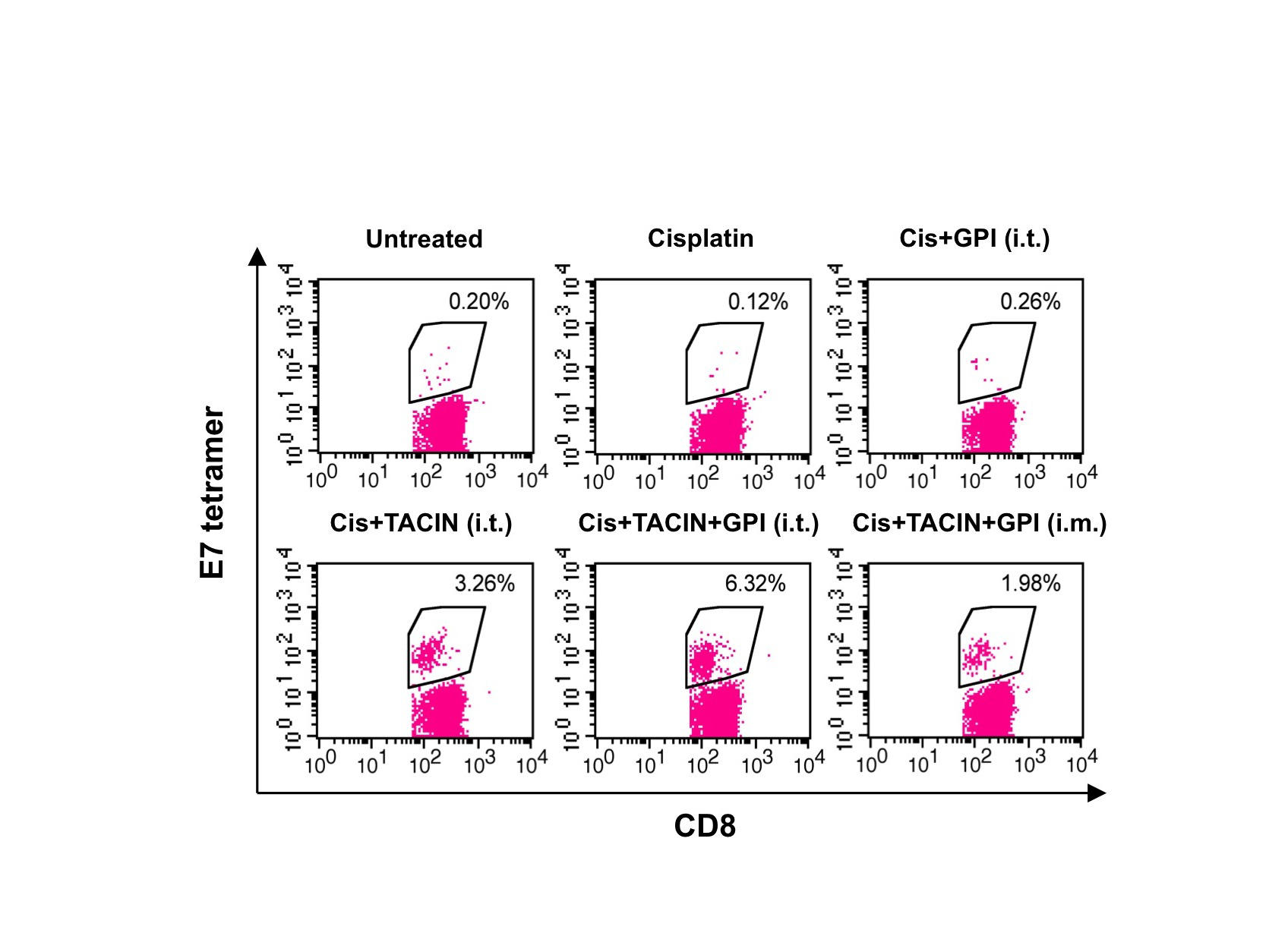

Supplement: S8 Fig — Representative of flow cytometry images of HPV16 E7-specific CD8+ T cell responses after chemotherapy and TA-CIN vaccination in TC-1 tumor bearing mice. The experiment was performed as illustrated in S7 Fig. One week after the last vaccination, PBMCs were prepared from the tumor-bearing mice, and stained with FITC-conjugated anti-mouse CD8a and PE-Conjugated HPV16 E7aa49-57 peptide loaded H-2Db tetramer. The data were acquired with FACSCalibur and analyzed with CellQuest (summarized in Fig. 6C). (TIF) [file pone.0116389.s008.tif]
